# ANXA6 Overexpression Causes Abnormal Decidual Macrophage-Trophoblast Crosstalk in Recurrent Spontaneous Abortion

**DOI:** 10.7150/ijbs.111791

**Published:** 2025-08-11

**Authors:** Xin Chen, Xue Yao Li, Xue Qin Ma, Yan Zhang, Qian Lin Song, Jing Yang

**Affiliations:** 1 Department of Obstetrics and Gynecology, Renmin Hospital of Wuhan University, Wuhan, Hubei, China.; 2 Reproductive Medical Center, Renmin Hospital of Wuhan University and Hubei Clinic Research Center for Assisted Reproductive Technology and Embryonic Development, Wuhan, Hubei, China.; 3 Department of Urology, Renmin Hospital of Wuhan University, Wuhan, Hubei, China.; 4 Central Laboratory, Renmin Hospital of Wuhan University, Wuhan, Hubei, China.

**Keywords:** Recurrent spontaneous abortion, ANXA6, Macrophage, NF-κB/ROS, Trophoblast, PI3K/AKT

## Abstract

ANXA6 is involved in numerous biological processes; however, its association with recurrent spontaneous abortion (RSA) remains poorly understood. In this study, we observed significant upregulation of ANXA6 expression in decidual macrophages from RSA patients. Functional analysis revealed that ANXA6 overexpression enhanced reactive oxygen species (ROS) generation and reduced mitochondrial membrane potential, thereby promoting pyroptosis and upregulating M1 macrophage polarization markers. Mechanistically, inhibition of NLRP3 rescued ANXA6 overexpression-induced elevation of M1 polarization and pyroptosis in macrophages. In addition, inhibition of ROS alleviated the decreased mitochondrial membrane potential, aggravated macrophage pyroptosis, and exacerbated inflammatory response, as well as the promoted macrophage M1 polarization caused by ANXA6 overexpression. Further mechanisms suggest that overexpression of ANXA6 in macrophages could promote the accumulation of mitochondrial ROS and inhibit mitochondrial membrane potential through the NF-κB signaling pathway, exacerbating macrophage pyroptosis and amplifying the resulting inflammatory response, thereby promoting macrophage M1 polarization. Besides, ANXA6 overexpressing macrophages showed an inhibitory effect on trophoblast function *in vitro*, a process mediated through TNF-α inhibition of the PI3K/AKT axis. Collectively, our study reveals that ANXA6 is a key mediator of immune dysregulation at the maternal-fetal interface in RSA patients.

## Introduction

Recurrent spontaneous abortion (RSA) is defined as two or more consecutive pregnancy losses prior to 28 weeks' gestation with the same partner, affecting approximately 1-5% of reproductive-aged women 1. The etiology of RSA is multifactorial, involving chromosomal abnormalities, genetic defects, uterine malformations, autoimmune disorders, and thrombophilia, etc 2, 3. Nevertheless, approximately half of RSA cases remain etiologically undefined, clinically referred to as unexplained RSA 4. RSA episodes impose significant physiological and psychological burdens on women of reproductive age. Therefore, it is highly significant to comprehensively investigate its pathogenesis and identify effective prevention and treatment strategies.

Emerging research highlights the critical role of decidual immune cells in maintaining maternal-fetal interface homeostasis. This heterogeneous population comprises both innate (natural killer cells, macrophages, dendritic cells, mast cells, myeloid-derived suppressor cells) and adaptive immune cells (T cells, B cells) 5-7. Decidual macrophages, representing the second most abundant leukocyte population at the maternal-fetal interface, play a crucial role in maintaining immune homeostasis during pregnancy. Dysregulation in their polarization or function may result in adverse pregnancy outcomes such as RSA 8.

Multiple studies have demonstrated the NLRP3 inflammasome's involvement in modulating macrophage polarization processes, i.e., when NLRP3 inflammasome is activated, it causes the precursor caspase-1 in the macrophage to undergo shearing to become activated caspase-1, and activated caspase-1 shears the precursors IL-1β and IL-18 to the mature form, which is released to the outside of the cell 9, 10. In addition, activated caspase-1 can also shear gasdermin D (GSDMD), leading to the pyroptosis and further exacerbating the inflammatory response 11, 12. However, whether pyroptosis of decidual macrophages contributes to RSA development requires further investigation.

ANXA6, the largest member of the annexin family of membrane-bound proteins, is highly expressed in most tissues 13-18. Emerging evidence indicates that ANXA6 participates in diverse biological processes 13-19. A recent investigation revealed significant positive correlations between ANXA6 expression levels and immune cell infiltration, including B cells, CD8^+^T cells, CD4^+^T cells, macrophages, neutrophils and dendritic cells 20. However, it remains unclear whether differential expression of ANXA6 influences to abnormal macrophage function and polarization and thus participates in the development and progression of RSA.

In the present study, we investigated the pathogenic role of ANXA6 in RSA. Our results demonstrated that ANXA6 promoted macrophage pyroptosis through the NF-κB/ROS signaling pathway, thereby facilitating the polarization of macrophages into the M1 phenotype. Furthermore, ANXA6-stimulated TNF-α secretion from macrophages suppresses PI3K/AKT signaling, consequently impairing trophoblast migration, invasion, and epithelial-mesenchymal transition (EMT). These collective findings identify ANXA6 as a promising therapeutic target for RSA management.

## Materials and Methods

### Clinical sample collection

With the approval of the Ethics Committee of our hospital, decidua samples were collected from 20 patients with recurrent spontaneous abortion (RSA group) and 20 patients with normal pregnancy (healthy control group, HC group). The baseline data of the two groups are shown in Table 1.

### RT-qPCR analysis

Total RNA was extracted from the samples using TRIzol reagent (15596026, Thermo Fisher Scientific Co., Ltd.). RNA was reverse transcribed into cDNA using a reverse transcription kit (RR036A, Takara, Shiga, Japan). The mRNA expression of target genes was detected by the TB Green Premix ExTaq II (TliRNaseH Plus) kit (RR420A, Takara). All primer sequences are provided in Table S1.

### Western blotting

Protein lysates were prepared using RIPA buffer (P0013B, Beyotime Biotechnology, Wuhan, China) and separated by SDS-PAGE. Subsequently, proteins were transferred to a PVDF membrane and blocked for 1 h. The membrane was then incubated overnight at 4 °C with primary antibodies, followed by a 1-h incubation with secondary antibodies. Specific details regarding the antibodies used are provided in Table S2.

### Co-immunoprecipitation (Co-IP)

Co-IP was performed using the Classic Magnetic Protein A/G Kit (YJ201, Epizyme Biotech, Shanghai, China) following the manufacturer's protocol. Cells were lysed, and supernatants were incubated overnight at 4 °C with specific antibodies. Protein A/G beads were added for 1 h at room temperature, followed by three washes. Immunoprecipitated complexes were analyzed by Western blot. Details regarding the antibodies used are provided in Table S2.

### Immunohistochemistry

Tissues were fixed in 4% paraformaldehyde (PFA), paraffin-embedded, and then sectioned. Following antigen retrieval, sections were blocked with 3% bovine serum albumin (BSA) /PBS (30 min) and incubated with primary antibodies at 4 °C for 24 h, and then with corresponding secondary antibodies for 1h. Staining was performed using DAB, counterstained with hematoxylin, and sections were sealed. Details regarding the antibodies used are provided in Table S2.

### Immunofluorescence

Cells: Samples were fixed with 4% PFA (15 min), permeabilized with 0.3% Triton X-100 (8 min) and blocked with 5% BSA (30 min). Primary antibodies were incubated overnight at 4 °C. After washing, samples were incubated with secondary antibodies (30 min, dark conditions), then stained with DAPI, and images were recorded.

Tissues: Deparaffinized sections underwent antigen retrieval, blocking (3% BSA, 30 min), and primary antibody incubation (4 °C, overnight). After PBST washes, secondary antibodies (50 min, dark) and tyramine-CY3/488 (20 min, dark) were applied. Nuclei were counterstained with DAPI and then examined under a microscope.

Detailed antibody information can be found in Table S2.

### Flow cytometry

Single cell suspensions were blocked for 10 minutes and then stained with fluorescent antibodies. Samples were analyzed using flow cytometry. Antibody details are provided in Table S2.

### Cell culture and intervention

HTR-8/SVneo (HTR8) and THP-1 cells were cultured in RPMI 1640 (Gibco) containing 10% fetal bovine serum and 1% penicillin-streptomycin (C0222, Beyotime Biotechnology), under standard conditions (37°C, 5% CO₂).

THP-1 cells were differentiated into M0 macrophages by treatment with 100 ng/mL phorbol 12-myristate 13-acetate (PMA, Sigma, USA) for 24 hours in complete RPMI 1640 medium.

Cell lines stably expressing specific genes were generated using an overexpression vector and lentiviral short hairpin RNA (shRNA) (GeneChem Co., Ltd., Shanghai, China). The overexpression vector was GV705, and the shRNA vector was GV112. Following 16-hour lentivirus incubation, stabilized transfected cells were selected via puromycin resistance screening.

Cell-cell interactions were studied using a Transwell co-culture system. Macrophages (upper chamber) were co-cultured with trophoblasts (lower chamber) without direct contact.

For the inhibition of pyroptosis, cells were treated with MCC950 (10 μmol/L, HY-12815, MCE, Shanghai, China), an NLRP3 inhibitor.

For inhibition of ROS, cells were treated with N-acetylcysteine (NAC) (4 mmol/L, HY-B0215, MCE), a specific ROS inhibitor.

Inhibition of the NF-κB pathway was achieved by treating cells with an NF-κB inhibitor [pyrrolidinedithiocarbamate ammonium (PDTC) (10 μmol/L, HY-18738, MCE)].

For TNF-α receptor blockade, cells were treated with infliximab (10 μg/mL, HY-P9970, MCE)].

For activation of the PI3K signaling pathway, the PI3K activator 740 Y-P (50 μg/mL, HY-P0175, MCE)] was used to treat cells.

### ROS

Intracellular ROS levels were quantified using the DCFH-DA fluorescent probe (CA1420, Solarbio, Beijing, China). Following the manufacturer's protocol, cells were loaded with DCFH-DA in serum-free medium (37 °C, 30 min, dark conditions).

### Mitochondrial membrane potential assay

Mitochondrial membrane potential was analyzed using the JC-1 fluorescent probe (M8650, Solarbio). Briefly, cells were incubated with JC-1 solution (1 mL) at 37°C for 20 min (dark conditions), followed by two buffer washes. Fluorescence signals were captured after replacing with fresh culture medium.

### TUNEL

TUNEL assay (C1088, Beyotime) was performed to assess apoptosis. After fixation, cells were permeabilized (0.3% Triton X-100, 5 min), incubated with TUNEL reaction mix (50 μL, 37 °C, 60 min, dark). Finally, the samples were observed.

### Enzyme-linked immunosorbent assay (ELISA)

Cytokine levels (IL-18(CSB-E07450h, Cusabio Wuhan, China), IL-1β (CSB-E08053h, Cusabio), IL-6(CSB-E04638h, Cusabio), IL-12(CSB-E04599h, Cusabio), IL-23(CSB-E08461h, Cusabio), and TNF-α (CSB-E04740h, Cusabio)) were determined using ELISA kits according to the manufacturer's protocol. Samples and standards were incubated with the provided reagents, and absorbance was determined using a microplate reader.

### Caspase-1 activity

Caspase-1 enzymatic activity was determined using the Caspase-1 Activity Assay Kit (C1102, Beyotime) following the manufacturer's protocol. The samples were analyzed, and the absorbance was measured at a wavelength of 405 nm.

### Wound healing assay

Cells were cultured until they reached 90% confluence. Linear wounds were generated using sterile 200 μL pipette tips, and wound healing was monitored at 0 and 48 h using light microscopy.

### Transwell invasion assay

Matrigel-coated (1:9) upper chambers were seeded with serum-free suspended cells, while the lower chamber contained 10% FBS culture medium. After 48h of culture, cells were methanol-fixed, crystal violet-stained, and imaged using light microscopy.

### Animal experiments

*In vivo* experiments were conducted using CBA/J females, DBA/2 males and BALB/c male mice. Four weeks prior to mating, female mice were exposed to intravenous injections of adeno-associated virus (AAV)9, which carries small hairpin RNA against ANXA6 (AAV-sh-ANXA6), the ANXA6 coding sequence (AAV-OE-ANXA6), or their corresponding negative control (AAV-sh-Ctrl or AAV-OE-Ctrl) (Genechem, Shanghai). CBA/J female mice were mated with BALB/c males (normal pregnancy, NP) or DBA/2 males (abortion-prone pregnancy, AP). On day 11.5 of pregnancy, mice were euthanized to determine embryo resorption rates and collect placental tissues.

### Statistical analysis

All data were presented as mean ± standard deviation (SD) from at least three independent experiments. For normally distributed data, Student's t-test or one-way ANOVA was used. If data were not normally distributed, the Kruskal-Wallis test was employed. *P* values < 0.05 were considered statistically significant.

## Results

### ANXA6 expression is notably elevated in decidual macrophages of RSA patients

**Figure 1:**
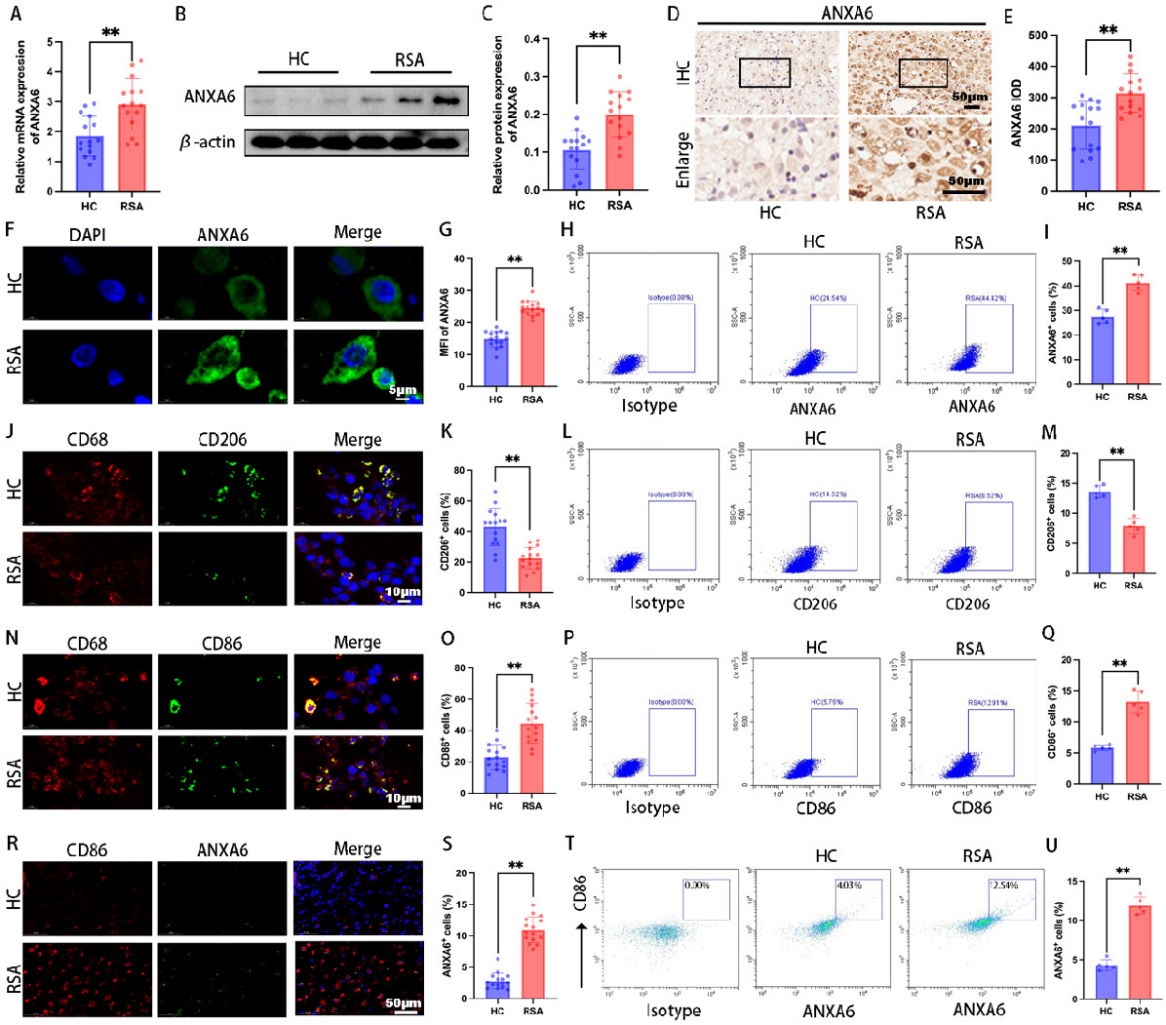
** ANXA6 expression is significantly upregulated in decidual macrophages of RSA patients.** ANXA6 expression in decidual tissues from HC and RSA patients (n=15) was assessed by **(A)** qPCR, **(B-C)** Western blotting, **(D-E)** immunohistochemistry, and **(F-G)** immunofluorescence, and the statistical value of ANXA6 was analyzed. **(H)** Representative flow cytometry analysis of ANXA6 expression in decidual tissue from HC and RSA patients (n=5). **(I)** The proportion of ANXA6⁺ cells in total decidual tissue (n=5). **(J)** CD68 and CD206 co-localization in the decidual tissue of HC and RSA patients (n=15). **(K)** Statistical value of the percentage of CD206^+^ cells in the CD68^+^ cells of HC and RSA patients (n=15). **(L-M)** Flow cytometry results of CD206 expression in decidual tissue and data show the percentage of CD206^+^ cells in the total decidual tissue of HC and RSA patients (n=5). **(N)** CD68 and CD86 co-localization in the decidual tissue of HC and RSA patients (n=15). **(O)** The percentage of CD86^+^ cells in the CD68^+^ cells of HC and RSA patients (n=15). **(P-Q)** Flow cytometry results of CD86 expression in decidual tissue and data show the percentage of CD86^+^ cells in the total decidual tissue of HC and RSA patients (n=5). **(R)** CD86 and ANXA6 co-localization in the decidual tissue of HC and RSA patients (n=15). **(S)** ANXA6^+^/CD86^+^ cells in HC and RSA patients (n=15). **(T-U)** Flow cytometry results of ANXA6 expression in CD86^+^ cells and data show the ANXA6^+^/CD86^+^ cells in HC and RSA patients (n=5). Student's t-test was used to assess differences between the two groups. **P < 0.01. IOD: Integrated Optical Density; MFI: Mean Fluorescence Intensity.

To investigate the role of ANXA6 in RSA, decidual tissue samples were collected RSA patients and healthy pregnant women (healthy control group, HC patients). The qPCR and Western blot assays revealed significantly higher ANXA6 expression in RSA samples (Fig.1A-C). Further examination of ANXA6 expression was performed using immunohistochemical and immunofluorescence staining. Consistent with qPCR and Western blotting findings, the results demonstrated that ANXA6 expression was significantly elevated in the decidual tissues of RSA patients compared with those of HC patients (Fig. 1D-G). Besides, flow cytometry results showed that ANXA6 expression in the decidual tissue of RSA patients was significantly higher than in HC patients (Fig. 1H-I). Immunofluorescence double-labeling and flow cytometry revealed a significant decrease in CD206^+^ macrophages and an increase in CD86^+^ macrophages in RSA decidual tissues (Fig. 1J-Q). Furthermore, ANXA6 expression in CD86^+^ macrophages was significantly higher in RSA patients than in HC patients (Fig. 1R-U). These data collectively indicate that ANXA6 expression is significantly elevated in the decidual macrophages of RSA patients.

### ANXA6 overexpression promotes M1 macrophage polarization

**Figure 2:**
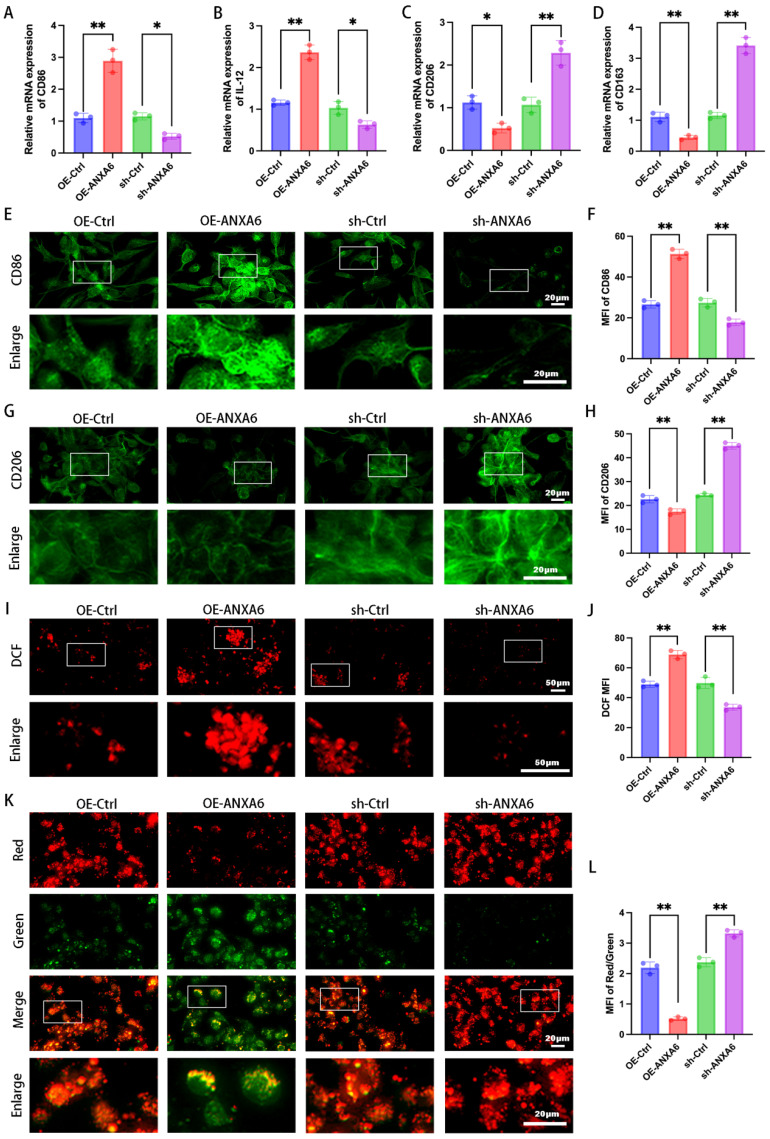
** ANXA6 overexpression promotes M1 macrophage polarization.** M0 macrophage differentiation was induced in THP-1 cells using 100 ng/mL PMA for 24 h, immediately following the use of ANXA6 overexpression and knockdown lentiviral vectors as well as virus-negative interventions in M0 cells. **(A-D)** qPCR was performed to analyze CD86, IL-12, CD206, and CD163 mRNA levels (n=3). **(E)** Fluorescence images of CD86 (n=3). **(F)** Quantitative values of CD86 mean fluorescence intensity (MFI) (n=3). **(G)** Fluorescence images of CD206 (n=3). **(H)** Quantitative values of CD206 MFI (n=3). **(I-J)** Intracellular ROS levels were assessed using DCFH-DA staining followed by immunofluorescence analysis, with MFI quantified. (n=3). **(K)** Representative images of mitochondrial membrane potential (MMP) using fluorescent probe JC-1 (n=3). **(L)** Statistical results of the MFI of MMP (n=3). One-way ANOVA was used to compare differences between multiple groups. *P < 0.05, **P < 0.01.

Given the significant increase in ANXA6 expression within the decidual macrophages of patients with RSA, we hypothesized that ANXA6 overexpression may be related to the abnormal polarization of decidual macrophages. To investigate the role of ANXA6 in RSA and macrophage polarization, THP-1 cells were first differentiated into M0 macrophages using PMA. The M0 macrophages were then subjected to ANXA6 overexpression or knockdown via lentiviral transduction, with successful modulation of ANXA6 expression confirmed (Fig. S1A-D). Next, we evaluated macrophage polarization by quantifying the expression of M1 markers (CD86 and IL-12) and M2 markers (CD206 and CD163) via qPCR, as well as analyzing inflammatory cytokine secretion using ELISA. The results demonstrated that the elevated expression of ANXA6 was accompanied by increased expression levels of M1 phenotypic markers and decreased expression levels of M2 phenotypic markers (Fig. 2A-D), while promoting the secretion of inflammatory cytokines (Fig. S2A). These findings suggest that ANXA6 promotes M1 polarization while suppressing M2 polarization. To further confirm these observations, we measured the expression of CD86 and CD206 by immunofluorescence and flow cytometry, and found that ANXA6 overexpression significantly upregulated CD86 while downregulating CD206 (Fig. 2E-H and Fig. S2B-E). Together, these results demonstrate that ANXA6 plays a crucial role in driving M1 polarization. Previous research has established that intracellular ROS production and reduction of mitochondrial membrane potential in macrophages are closely related to macrophage M1 polarization 21. Hence, we investigated the alterations in these parameters within our macrophage groups. The results indicated that ANXA6 overexpression led to a notable elevation in ROS levels (Fig. 2I-J) and a reduction in mitochondrial membrane potential (Fig. 2K-L) in macrophages. These data suggest that ANXA6 regulates macrophage polarization and is strongly associated with mitochondrial ROS.

### ANXA6 overexpression promotes macrophage pyroptosis

**Figure 3:**
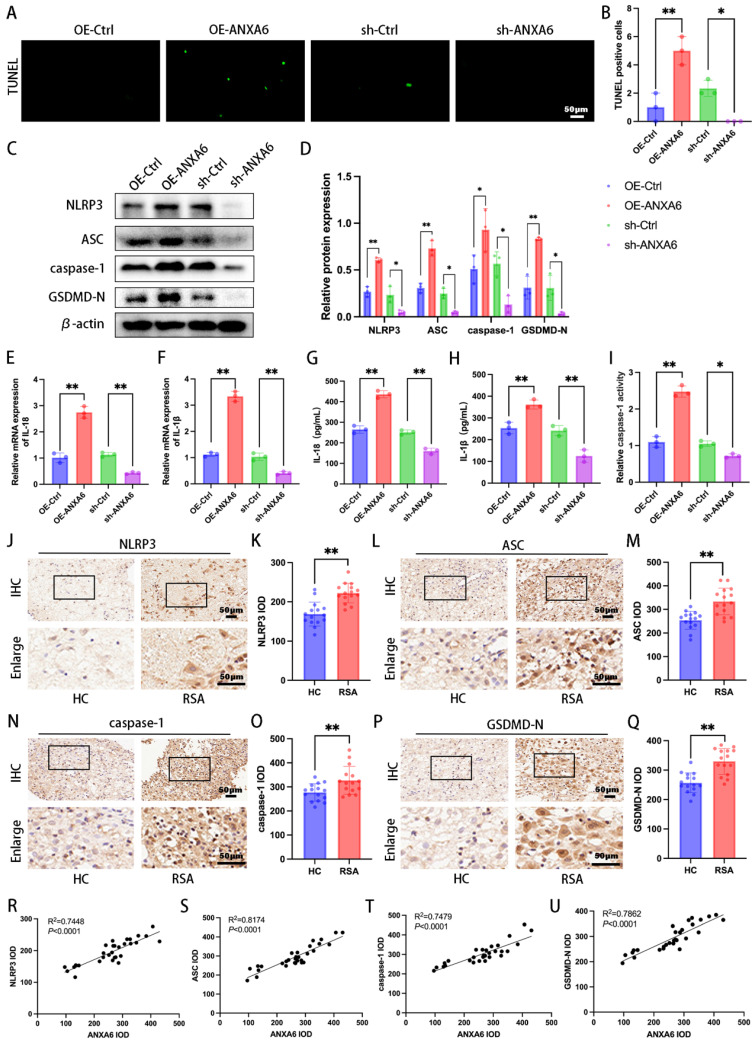
** ANXA6 promotes macrophage pyroptosis. (A)** The pyroptosis rate was assessed by TUNEL staining (n=3). **(B)** The number of TUNEL-positive cells was quantified (n=3). **(C-D)** The protein levels of NLRP3, ASC, caspase-1, and GSDMD-N were analyzed by Western blotting, and the statistical value was analyzed (n=3). **(E-F)** qPCR and **(G-H)** ELISA analyses revealed IL-1β and IL-18 expression levels (n=3). **(I)** The activity of caspase-1 (n=3). **(J-Q)** Immunohistochemical staining revealed NLRP3, ASC, caspase-1, and GSDMD-N expression in the decidual tissue from HC and RSA patients (n=15) and the IOD was analyzed. **(R-U)** Simple linear regression analysis revealed significant associations between ANXA6 and NLRP3, ASC, caspase-1, or GSDMD-N in decidual tissue (n=30). Student's t-test and one-way ANOVA were employed for comparisons between two groups and multiple groups, respectively. *P < 0.05, **P < 0.01.

Aberrant accumulation of mitochondrial ROS has been shown to activate NLRP3 inflammasome and further induce pyroptosis 22. Our experimental results suggest that ANXA6 promotes mitochondrial damage and ROS overproduction. To further determine whether the differential expression of ANXA6 in macrophages is associated with pyroptosis, each group of cells was first examined by TUNEL staining, which revealed markedly increased pyroptosis in ANXA6-overexpressing macrophages versus control cells (Fig. 3A-B). Following this, pyroptosis-related proteins (NLRP3, ASC, caspase-1, and GSDMD-N) were analyzed by Western blotting. The results indicated that ANXA6 overexpression in macrophages promoted the expression of these pyroptosis markers, while knockdown of ANXA6 inhibited the expression of these pyroptosis markers (Fig. 3C-D). The expression of inflammatory cytokines IL-1β and IL-18 was detected by qPCR. Our findings demonstrated that overexpression of ANXA6 promoted the expression of IL-1β and IL-18, while knockdown of ANXA6 suppressed the expression of IL-1β and IL-18 (Fig. 3E-F).

Subsequently, the concentrations of IL-1β and IL-18 were quantified using ELISA kits. The results showed that compared to the control group, ANXA6 overexpression significantly increased IL-1β and IL-18 levels, whereas ANXA6 knockdown markedly decreased both inflammatory cytokines (Fig. 3G-H). Similarly, caspase 1 activity was significantly increased in the ANXA6 overexpression group but markedly suppressed in the ANXA6 knockdown group (Fig. 3I). We further validated whether the expression of pyroptosis markers was enhanced in the decidual tissue of RSA patients. The expression levels of all these pyroptosis markers were significantly higher in the decidual tissues of RSA patients than in HC patients (Fig. 3J-Q). Furthermore, simple linear regression analysis was performed to assess the relationship between ANXA6 expression and pyroptosis markers in decidual tissues. The results indicated a positive correlation between ANXA6 expression and pyroptosis markers in the decidual tissue (Fig. 3R-U). Taken together, the above data demonstrate that ANXA6 overexpression promotes macrophage pyroptosis.

### ANXA6 promotes M1 macrophage polarization by exacerbating macrophage pyroptosis

**Figure 4:**
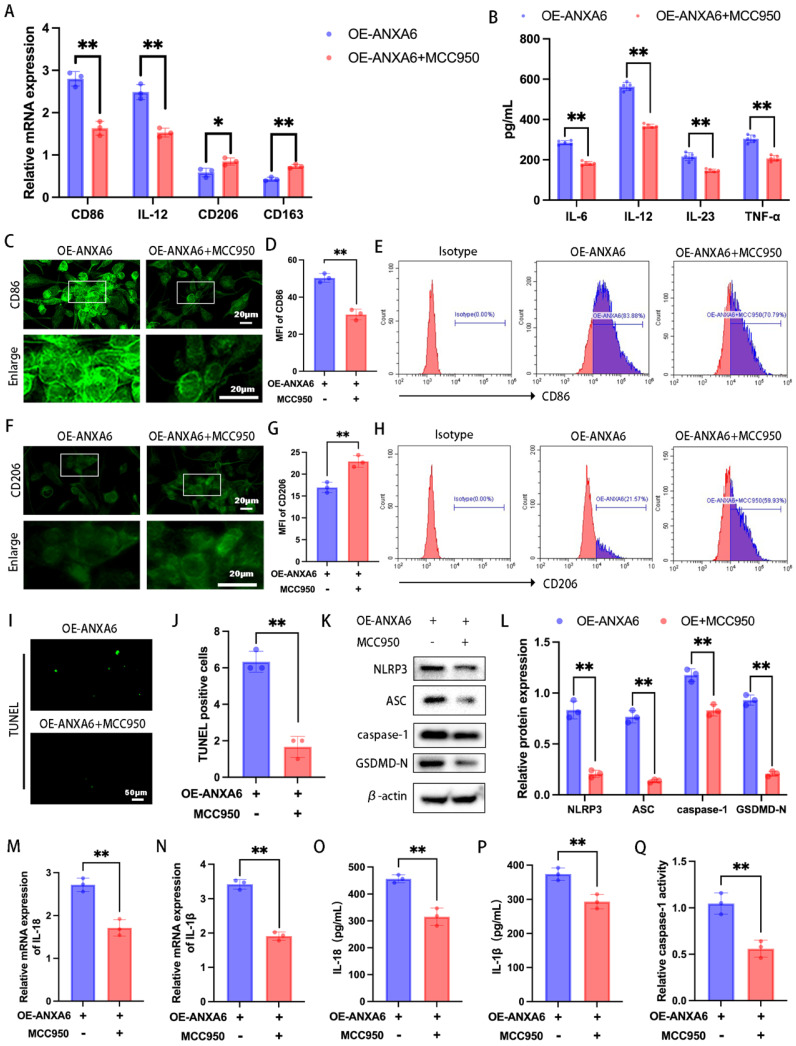
** ANXA6 promotes the inflammatory phenotype of macrophages by exacerbating macrophage pyroptosis. (A)** qPCR was performed to analyze CD86, IL-12, CD206, and CD163 mRNA levels (n=3). **(B)** Detection of inflammatory cytokine secretion by ELISA kits (n=5). **(C)** Fluorescence images of CD86 (n=3). **(D)** Quantitative values of the MFI of CD86 (n=3). **(E)** Representative flow cytometry results of CD86 (n=3). **(F)** Representative fluorescence images of CD206 (n=3). **(G)** Quantification of the MFI of CD206 (n=3). **(H)** Representative flow cytometry results of CD206 (n=3). **(I)** Examination of pyroptosis of each group of cells by TUNEL staining (n=3). **(J)** The number of TUNEL-positive cells was quantified (n=3). **(K-L)** NLRP3, ASC, caspase-1, and GSDMD-N protein levels were assessed by Western blotting (n=3), and the statistical value was analyzed. **(M-N)** qPCR analysis revealed mRNA expression of inflammatory cytokines IL-1β and IL-18 (n=3). **(O-P)** Protein levels of IL-1β and IL-18 were quantified by ELISA (n=3). **(Q)** The activity of caspase-1 (n=3). Student's t-test was used to assess differences between the two groups. *P < 0.05, **P < 0.01.

To further determine the role of ANXA6-induced pyroptosis in influencing inflammatory macrophage polarization, we treated in macrophages with MCC950 (an NLRP3 inhibitor). The expression levels of M1 phenotypic markers and M2 phenotypic markers were detected via qPCR and inflammatory cytokine secretion profiles were detected by ELISA. The results demonstrated that MCC950 reversed the increase in the expression levels of M1 phenotypic markers and the secretion of inflammatory cytokines and the reduction in the expression levels of M2 phenotypic markers induced by ANXA6 overexpression (Fig. 4A-B). However, in the absence of ANXA6 overexpression, MCC950 yielded no significant effect (Fig. S3A-B). To validate this finding, we further measured the expression of CD86 and CD206 by immunofluorescence and flow cytometry, and similarly found that MCC950 reversed the ANXA6-induced promotion of CD86 expression and the ANXA6-induced inhibition of CD206 expression (Fig. 4C-H, Fig. S3E, Fig. S3J). However, MCC950 yielded no effect in the absence of ANXA6 overexpression (Fig. S3C-D, Fig. S3F-I, Fig. S3K-L). Concurrently, we examined changes of pyroptosis in macrophages, and interestingly, the results showed that inhibition of NLRP3 after MCC950 treatment reduced ANXA6-activated pyroptosis in macrophages (Fig. 4I-Q); however, in the absence of ANXA6 overexpression, MCC950 yielded no significant effect (Fig. S3M-U). These results collectively suggest that ANXA6 promotes the inflammatory phenotype of macrophages by exacerbating macrophage pyroptosis.

### NF-κB/ROS signaling axis is involved in regulating ANXA6-mediated macrophage M1 polarization and pyroptosis

**Figure 5:**
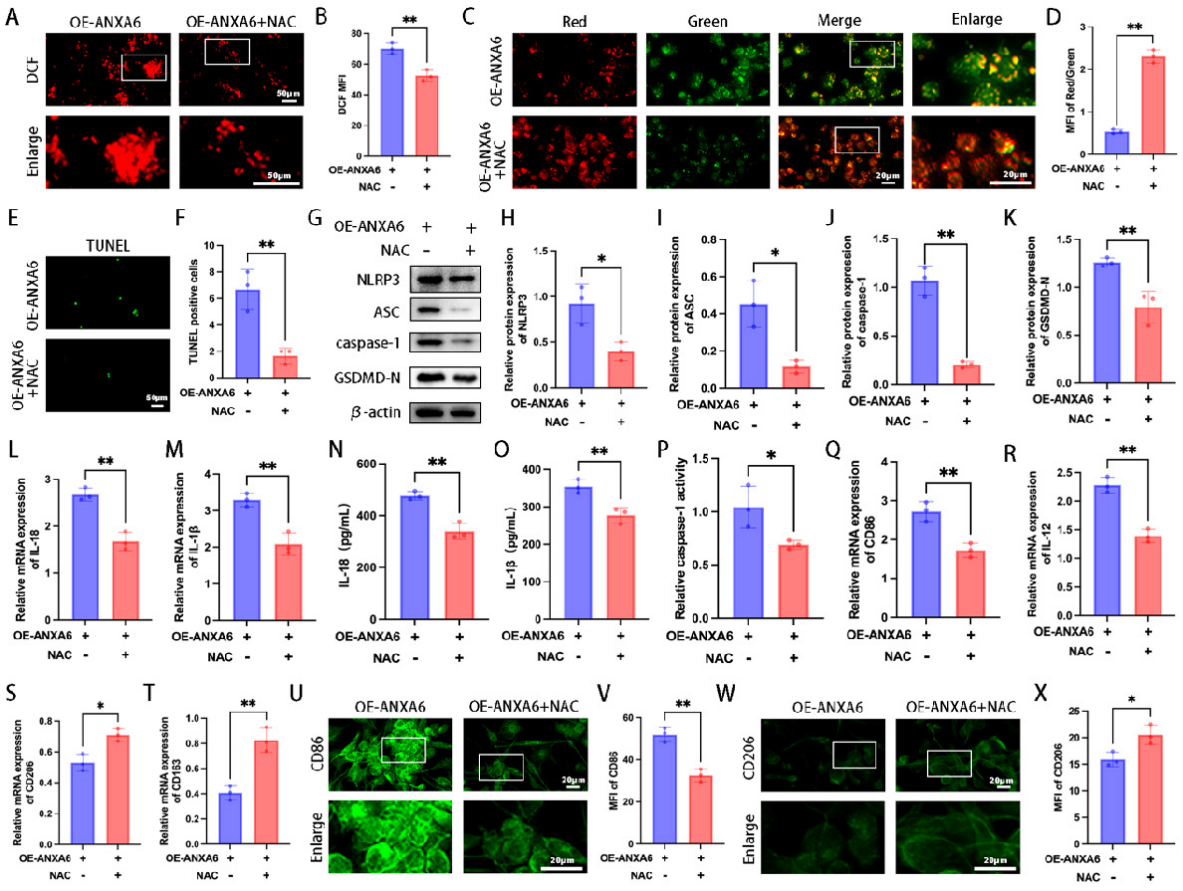
** ANXA6 promotes macrophage M1 polarization and pyroptosis by facilitating ROS production. (A-B)** Macrophage cells stained with DCFH-DA were detected by immunofluorescence and MFI was calculated (n=3). **(C)** Representative images of MMP using fluorescent probe JC-1 (n=3). **(D)** Statistical results of the MFI of MMP (n=3). **(E)** Examination of pyroptosis in each group of macrophage cells by TUNEL staining (n=3). **(F)** The number of TUNEL-positive cells was quantified (n=3). **(G-K)** Western blot analysis confirmed expression of pyroptosis-related proteins (NLRP3, ASC, caspase-1, and GSDMD-N) (n=3), and the statistical value was analyzed. **(L-M)** qPCR analysis revealed mRNA expression of inflammatory cytokines IL-1β and IL-18 (n=3). **(N-O)** Protein levels of IL-1β and IL-18 were quantified by ELISA (n=3). **(P)** The activity of caspase-1 (n=3). **(Q-T)** CD86, IL-12, CD206, and CD163 were assessed by qPCR (n=3). **(U)** Representative fluorescence images of CD86 (n=3). **(V)** Quantitative values of the MFI of CD86 (n=3). **(W)** Representative fluorescence images of CD206 (n=3). **(X)** Quantitative values of the MFI of CD206 (n=3). Student's t-test was used to assess differences between the two groups. *P < 0.05, **P < 0.01.

Our study findings demonstrated that ANXA6 boosted intracellular ROS production and attenuated mitochondrial membrane potential in macrophages. Given the established critical role of ROS in NLRP3 inflammasome activation 23, we further evaluated whether ROS is involved in NLRP3 inflammasome activation and pyroptosis, and its subsequent influence on macrophage polarization. The results showed that ROS triggered by ANXA6 was significantly inhibited by the ROS scavenger NAC (Fig. 5A-B). Meanwhile, NAC restored the mitochondrial membrane potential inhibited by ANXA6 overexpression (Fig. 5C-D). Further analysis of cellular pyroptosis across various experimental groups indicated that NAC inhibited pyroptosis promoted by ANXA6 overexpression in macrophages (Fig. 5E-P), indicating that ANXA6-induced ROS accumulation activates pyroptosis. In addition, NAC inhibited M1 macrophage polarization induced by ANXA6 overexpression in macrophages (Fig. 5Q-X and Fig. S4A-E). Overall, these data underscore the critical role of ROS in ANXA6-triggered M1 macrophage polarization and pyroptosis.

**Figure 6:**
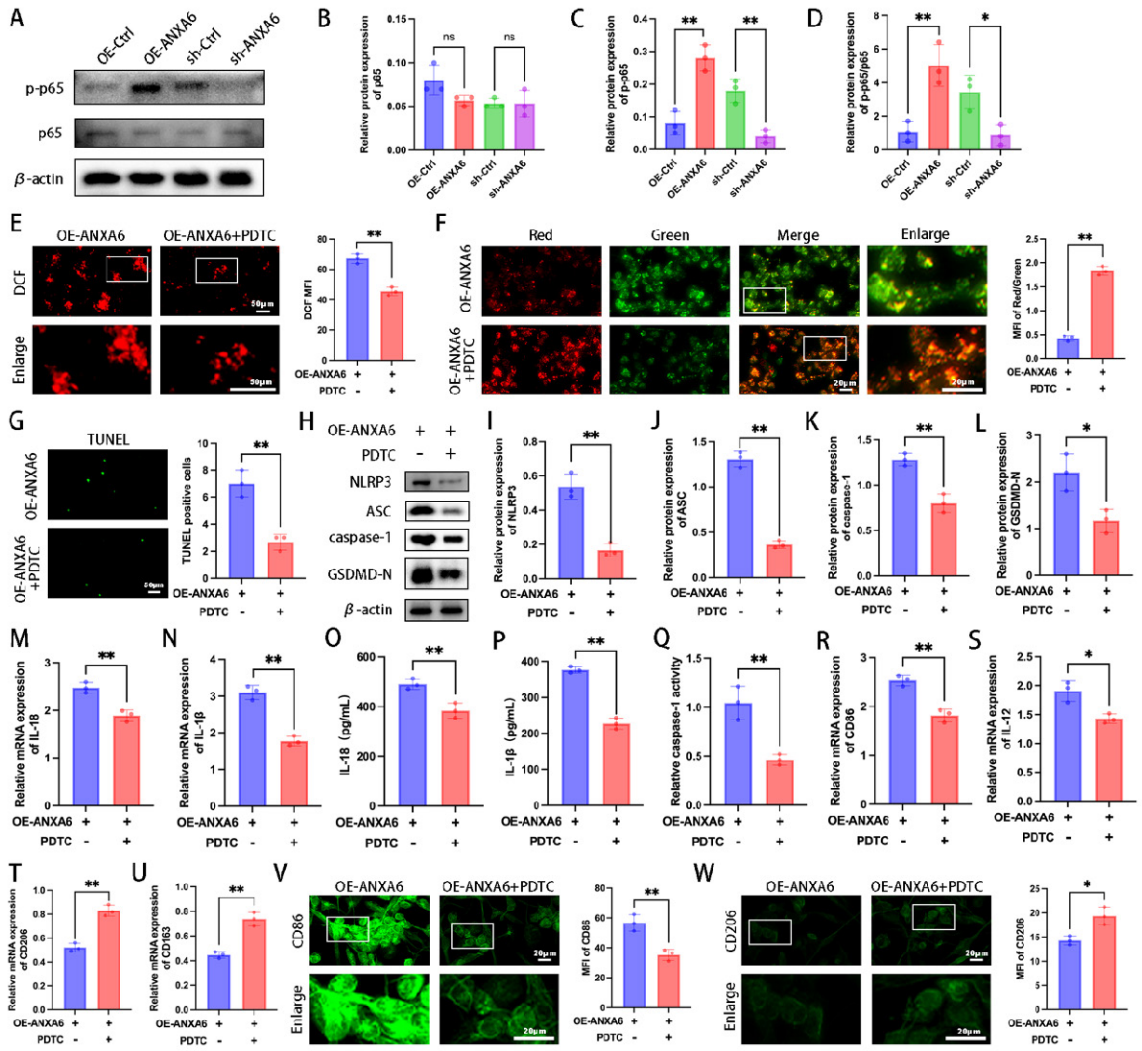
** NF-κB signaling mediates ANXA6-induced macrophage M1 polarization and pyroptosis. (A)** Detection of p65 and p-p65 expression using Western blotting (n=3). **(B-D)** Quantitative values of p65 and p-p65 expression (n=3). **(E)** Macrophage cells stained with DCFH-DA were detected by immunofluorescence and MFI was calculated (n=3). **(F)** Representative images of MMP and statistical results of MMP (n=3). **(G)** Examination of pyroptosis in each group of macrophage cells by TUNEL staining and statistical value of the number of TUNEL-positive cells (n=3). **(H-L)** Western blot analysis revealed expression of pyroptosis markers (NLRP3, ASC, caspase-1, and GSDMD-N) (n=3), and the statistical value was analyzed. **(M-N)** qPCR analysis revealed IL-1β and IL-18 mRNA expression in macrophages (n=3). **(O-P)** IL-1β and IL-18 protein levels were quantified by ELISA (n=3). **(Q)** The activity of caspase-1 (n=3). **(R-U)** The mRNA expression levels of CD86, IL-12, CD206, and CD163 (n=3). **(V)** Fluorescence images of CD86 and quantitative values of the MFI of CD86 (n=3). **(W)** Representative fluorescence images of CD206 and quantitative values of the MFI of CD206 (n=3). Student's t-test and one-way ANOVA were employed for comparisons between two groups and multiple groups, respectively. *P < 0.05, **P < 0.01, ns: not significant.

Previous studies have established that the NF-κB signaling pathway plays a regulatory role in pyroptosis 24. To elucidate the mechanistic link between ANXA6 and macrophage M1 polarization/pyroptosis, we analyzed the NF-κB signaling pathway. The results showed that ANXA6 increased the expression of p-p65/p65(Fig. 6A-D). Furthermore, we demonstrated that ANXA6 could directly interact with NF-κB and enhance its phosphorylation (Fig. S5A-B). Next, to further explore the role of the NF-κB signaling pathway in the impact of ANXA6 on macrophages, macrophages were treated with the NF-κB inhibitor PDTC. The results indicated that PDTC treatment significantly reduced ROS production compared to untreated controls (Fig. 6E). Besides, PDTC treatment significantly increased mitochondrial membrane potential compared to untreated controls (Fig. 6F). Subsequently, the alterations in macrophage pyroptosis were investigated across each group. The results demonstrated that pyroptosis was notably suppressed in macrophages following the addition of PDTC compared to untreated controls (Fig. 6G-Q). Finally, we further verified the phenotypes of macrophages. The results revealed that PDTC treatment significantly suppressed the M1 phenotype while promoting M2 macrophage polarization compared to untreated controls (Fig. 6R-W and Fig. S6A-E). In conclusion, these results suggest that ANXA6 regulates macrophage polarization and pyroptosis through the NF-κB/ROS signaling axis.

### ANXA6 overexpressing macrophages inhibit trophoblast function by inhibiting the PI3K/AKT axis via TNF-α

**Figure 7:**
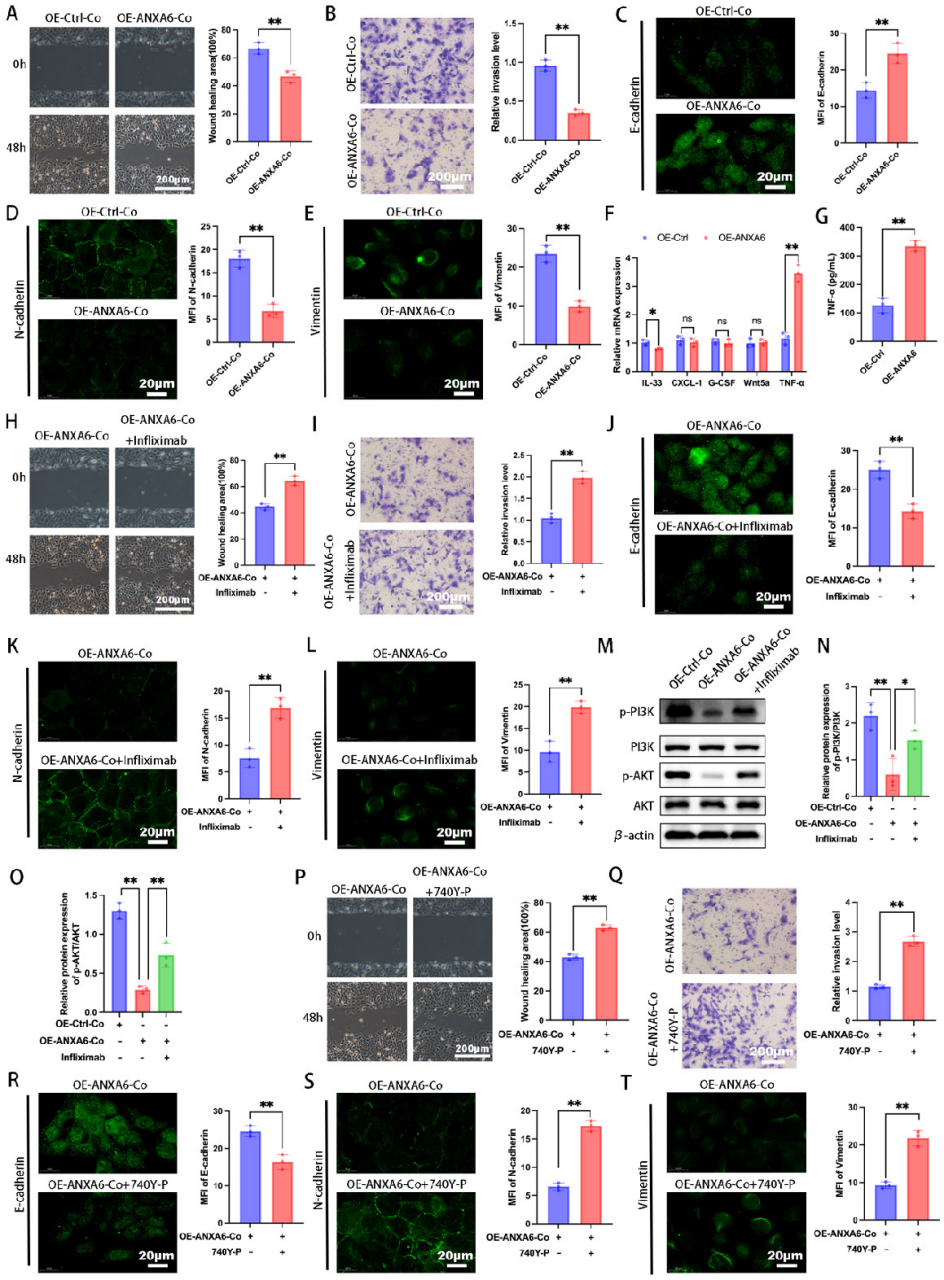
** Macrophages overexpressing ANXA6 inhibit the PI3K/AKT axis via TNF-α thereby inhibiting trophoblast function. (A)** Quantitative values of migration ability and migration ability of HTR8 cells were detected by wound healing assays (n=3). **(B)** Quantitative values of invasive ability and invasive capacity of HTR8 cells detected by transwell assays (n=3). **(C-E)** Immunofluorescence analysis revealed the expression of E-cadherin, N-cadherin, and vimentin in each group and quantitative values of the MFI (n=3). **(F)** Differences in the expression of IL-33, CXCL-1, G-CSF, Wnt5a, and TNF-α in macrophages from ANXA6 overexpression and control groups were examined by qPCR (n=3). **(G)** TNF-α protein levels were quantified by ELISA (n=3). **(H)** Quantitative values of migration ability and migration ability of HTR8 cells were assessed using wound healing assays (n=3). **(I)** Quantitative values of invasive ability and invasive capacity of HTR8 cells detected by transwell assays (n=3). **(J-L)** E-cadherin, N-cadherin, and vimentin were measured by immunofluorescence and quantitative values of the MFI (n=3). **(M-O)** Western blot analysis was performed to evaluate the expression of p-PI3K, PI3K, p-AKT and AKT (n=3), and the statistical value was analyzed. **(P-T)** Trophoblast migration, invasion, and EMT were detected after activation of the PI3K pathway using 740 Y-P (PI3K agonist) (n=3). Student's t-test and one-way ANOVA were employed for comparisons between two groups and multiple groups, respectively. *P < 0.05, **P < 0.01, ns: not significant.

Our previous work demonstrated bidirectional communication between trophoblasts and decidual macrophages during pregnancy 4, 25. Abnormal trophoblast function is a known contributor to pathological pregnancies, such as RSA 26. To further investigate whether the differential expression of ANXA6 in macrophages affects trophoblast function, macrophages and trophoblasts were co-cultured indirectly using a Transwell system, with cells separated into upper and lower chambers, respectively. We first assessed the migratory and invasive abilities and showed that trophoblast cells in the ANXA6 overexpression group exhibited decreased migratory and invasive abilities compared with the control group (Fig. 7A-B). EMT is defined by polarized epithelial cells losing polarity and intercellular contraction and acquiring mesenchymal cell properties 27, 28. EMT pathway activation can reportedly promote cell invasion and migration 29. Besides, EMT progression is marked by downregulation of E-cadherin, accompanied by upregulation of N-cadherin and vimentin expression 27. Our immunofluorescence assay revealed that ANXA6 overexpression in macrophages induced E-cadherin upregulation and inhibited N-cadherin and vimentin in trophoblast cells (Fig. 7C-E). The above data suggest that ANXA6-overexpressing macrophages exert an inhibitory effect on trophoblast function.

Macrophages have been found to produce and secrete IL-33, CXCL-1, G-CSF, Wnt5a, and TNF-α to regulate trophoblast function 30-34. Subsequently, we further explored how the differential expression of ANXA6 in macrophages affects trophoblast function. The expression of these molecules in ANXA6 control and ANXA6 overexpression macrophages was initially examined by qPCR. The qPCR results demonstrated that in the ANXA6 overexpression group, the levels of IL-33 and TNF-α exhibited varying degrees of alterations, with the most prominent increase in TNF-α (Fig. 7F). Next, the supernatants were collected from macrophages in both the ANXA6 overexpression group and the control group, and measured TNF-α expression using an ELISA assay. In the ANXA6 overexpression group, the TNF-α level was significantly higher (Fig. 7G).

Subsequently, to further validate the role of TNF-α, trophoblasts co-cultured with macrophages were treated with or without infliximab, a TNF-α receptor blocker. Wound healing and Transwell assays demonstrated that infliximab treatment significantly enhanced trophoblast migration and invasion compared to the untreated group (Fig. 7H-I). To further assess macrophage-mediated effects on trophoblast function, immunofluorescence was performed to evaluate EMT marker expression. The results showed that infliximab-treated trophoblasts exhibited reduced E-cadherin alongside elevated N-cadherin and vimentin levels (Fig. 7J-L). Collectively, these findings suggest that ANXA6 overexpression in macrophages promotes TNF-α production and impairs trophoblast function.

Given that PI3K/AKT signaling represents a critical molecular pathway governing trophoblast function 35, 36, we further investigated whether ANXA6 overexpression in macrophages modulates this pathway. Western blot results revealed that macrophages with ANXA6 overexpression induced a reduction in the levels of p-PI3K and p-AKT proteins in trophoblast cells, in contrast to control cells (Fig. 7M-O). Nevertheless, infliximab reversed the inhibitory effect on p-PI3K and p-AKT protein expression in trophoblasts caused by ANXA6 overexpression in macrophages (Fig. 7M-O). To elucidate the role of the PI3K/AKT pathway, the PI3K agonist 740 Y-P was employed to activate this signaling cascade, followed by assessment of trophoblast migration, invasion, and EMT markers expression. The results showed that 740 Y-P reversed the adverse effects of ANXA6 overexpression in macrophages on trophoblast cells (Fig. 7P-T). These results confirmed that macrophages overexpressing ANXA6 inhibited trophoblast function by inhibiting the PI3K/AKT axis, a process mediated via TNF-α.

### ANXA6 is upregulated in the aborted mice

**Figure 8:**
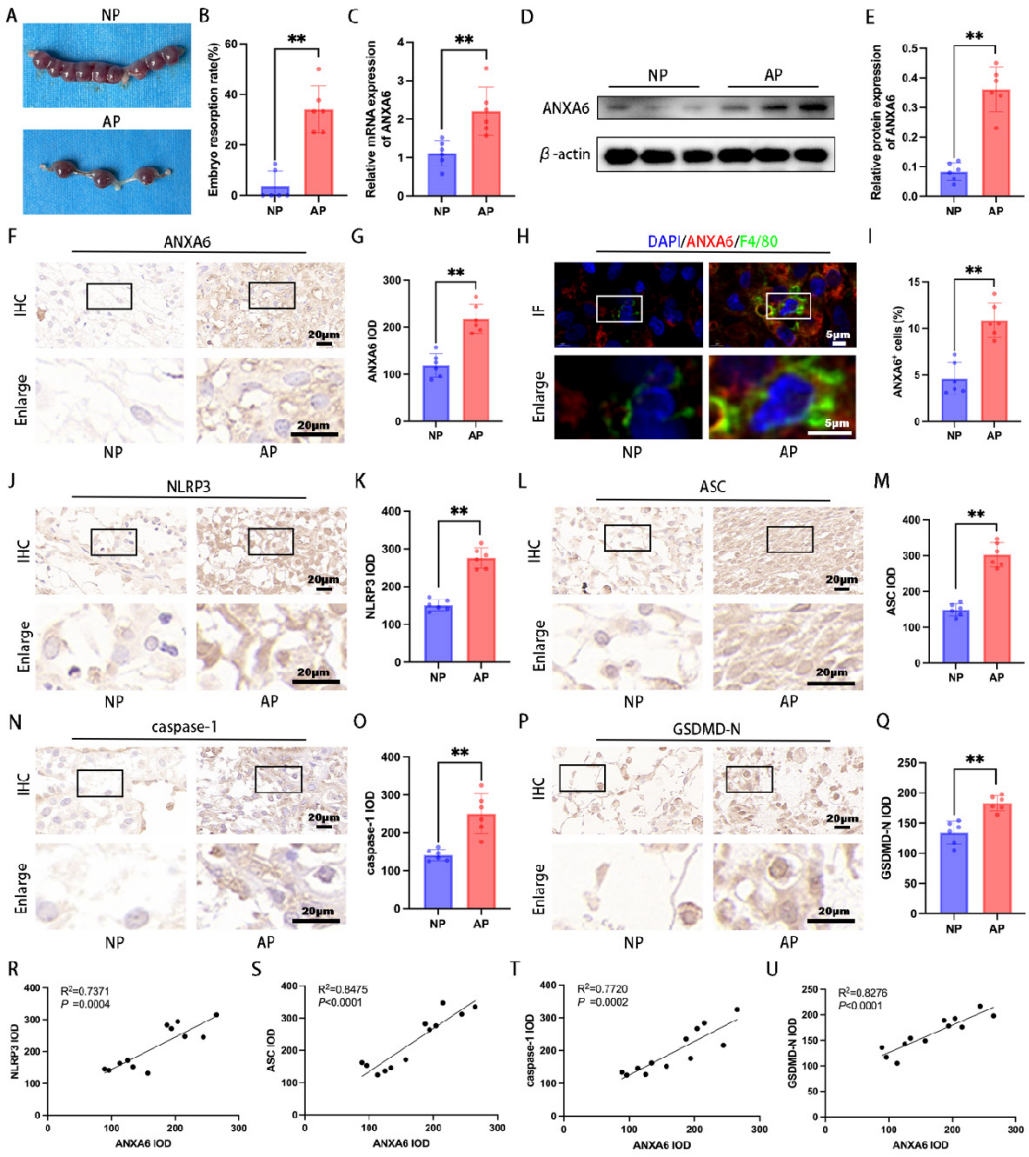
** ANXA6 is up-regulated at the placental interface of aborted mice and is strongly associated with pyroptosis. (A)** Typical macroscopic images of the uterus in NP and AP group mice (n=6). **(B)** Embryo resorption rate of NP and AP group mice (n=6). **(C)** The expression of ANXA6 in placental tissues of NP and AP group (n=6) mice were detected by qPCR. **(D-E)** Western blotting assay was used to verify the expression of ANXA6 in the placental tissues of NP and AP group mice (n=6), and the statistical value of ANXA6 was analyzed. **(F-G)** Immunohistochemical analysis was performed to assess ANXA6 expression in placental tissues of NP and AP group (n=6) mice and the IOD was analyzed. **(H)** Co-localization of ANXA6 and F4/80 in placental tissues of NP and AP group mice (n=6). **(I)** The percentage of ANXA6^+^ cells in the F4/80^ +^ cells of NP and AP group mice (n=6). **(J-Q)** Immunohistochemical analysis was used to detect the expression of pyroptosis markers (NLRP3, ASC, caspase-1, and GSDMD-N) in placenta tissues of NP and AP group mice (n=6), and the IOD was analyzed. **(R-U)** The association between ANXA6 and NLRP3, ASC, caspase-1 or GSDMD-N protein expression was investigated by simple linear regression analysis (n=12). Student's t-test was used to assess differences between the two groups. **P < 0.01.

To elucidate ANXA6's role in RSA, both NP and AP mouse models were employed for *in vivo* studies. The AP group exhibited significantly higher embryo resorption rates compared to the NP group (Fig. 8A-B). The qPCR and Western blot were employed to evaluate ANXA6 levels in placental tissues from both groups. The findings revealed that placental ANXA6 expression was significantly higher in the AP group compared to the NP group (Fig. 8C-E). Immunohistochemical analysis revealed stronger ANXA6 staining in AP decidua compared to the NP group (Fig. 8F-G). Furthermore, immunofluorescence double-labeling and flow cytometry analysis further confirmed that the number of ANXA6-positive cells within decidual macrophage populations was significantly higher in the AP group compared to the NP group (Fig. 8H-I and Fig. S7A-B). To investigate the underlying mechanism, immunohistochemical analysis revealed significantly elevated expression of pyroptosis markers in decidual tissues of AP mice compared to NP mice (Fig. 8J-Q). Besides, the correlation between ANXA6 expression and pyroptosis markers in mouse decidual tissues was examined. The experimental data revealed a significant positive correlation between ANXA6 expression levels and pyroptosis-related markers (Fig. 8R-U). Further studies revealed that overexpression of ANXA6 in normal pregnant mice resulted in increased embryo reabsorption (Fig. S8A-B), whereas knockdown of ANXA6 in abortion-prone mice alleviated embryo reabsorption (Fig. S8C-D). Overall, these results suggest that ANXA6 expression is upregulated at the placental interface in AP mice and is an essential factor in the pathogenesis of RSA.

## Discussion

**Figure 9:**
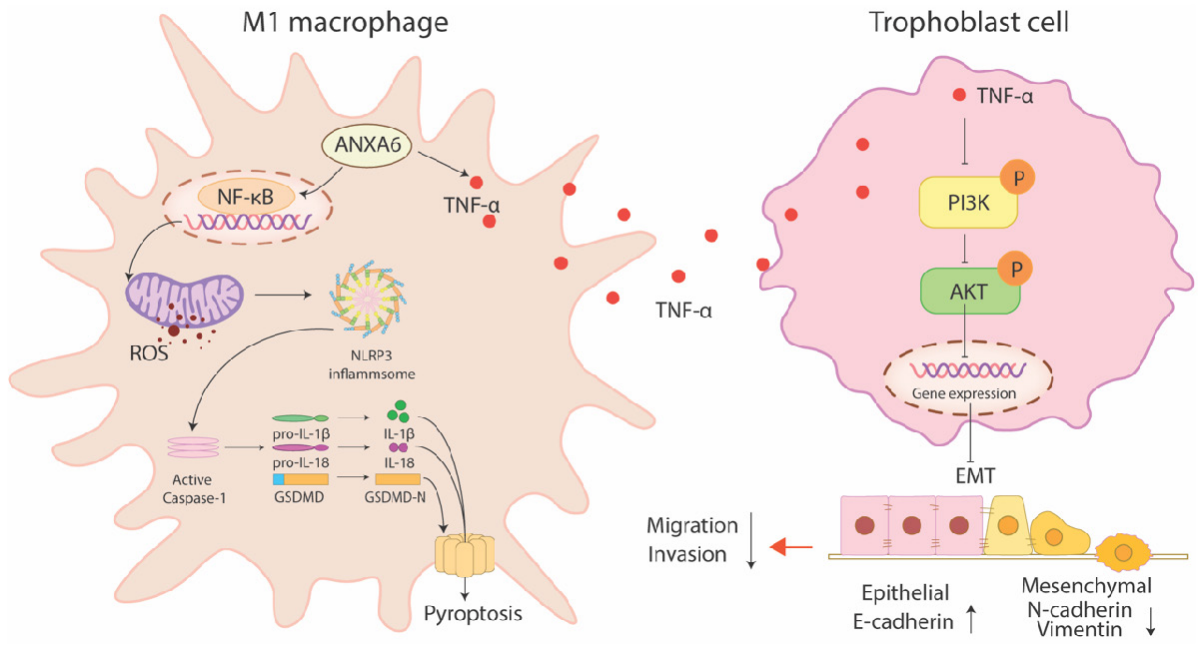
ANXA6-overexpressing macrophages promote macrophage pyroptosis via NF-κB/ROS signaling, which promotes macrophage M1 polarization. In addition, ANXA6-overexpressing macrophages inhibited trophoblast migration, invasion, and EMT by secreting TNF-α to suppress the PI3K/AKT pathway.

RSA not only damages physical health but also imposes far-reaching psychological and economic burdens on patients, making the study of its etiology and mechanism particularly important. The present study identified substantial upregulation of ANXA6 in decidual macrophages obtained from women with RSA. Overexpression of ANXA6 in macrophages promoted macrophage pyroptosis through NF-κB/ROS signaling via promoting the accumulation of mitochondrial ROS and inhibiting mitochondrial membrane potential, which in turn promoted macrophage M1 polarization. In addition, ANXA6-overexpressed macrophages showed an inhibitory effect on trophoblast migration, invasion, and EMT, and this effect was mediated through inhibition of the PI3K/AKT axis by TNF-α (Fig. 9).

RSA is a prevalent complication of pregnancy, with only about half of its etiological mechanisms clarified clinically 37-39. Most patients remain inadequately treated, often experiencing multiple pregnancy failures, thus leaving them physically and mentally exhausted in their pursuit of parenthood 1, 40. As the second most abundant leukocyte population at the maternal-fetal interface, decidual macrophages represent a critical component of placental immune cells and play an indispensable role in maintaining pregnancy-associated immune homeostasis 8. However, the precise factors governing decidual macrophage homeostasis remain largely undefined. Thus, identifying the underlying causes and exploring the associated pathogenic mechanisms are of paramount significance. The present study revealed a macrophage polarization imbalance in RSA, with decidual tissues exhibiting increased M1/M2 macrophage ratios compared to those in healthy pregnancies. Studies have shown that ANXA6 is associated with cell proliferation, survival, differentiation, inflammation, membrane repair, and viral infection 13-19. A study also revealed a marked positive correlation between ANXA6 expression and macrophage infiltration 20. Our results demonstrated that ANXA6 expression levels were remarkably elevated in the decidual tissue and decidual macrophages of RSA patients and aborted mice. In addition, *in vitro* cellular experiments further confirmed that ANXA6 could regulate macrophage M1 polarization by altering mitochondrial ROS.

Previous studies have linked M1 macrophage polarization to pyroptosis^41^, ^42^. It was also found that increased miR-3074-5p expression promoted macrophage M1 polarization and pyroptosis through the ERα/NLRP3 pathway and induced adverse pregnancy outcomes in mice 43. Our study revealed that ANXA6 promotes the inflammatory phenotype of macrophages by exacerbating macrophage pyroptosis. We further explored whether pyroptosis occurs in the decidual tissue of RSA patients and aborted mice. Immunohistochemistry analysis revealed significantly increased expression of pyroptosis markers in decidual tissues from both RSA patients and aborted mice, consistent with the literature 44. Furthermore, our study revealed a significant positive correlation between ANXA6 expression levels and pyroptosis markers in decidual tissues. Hence, these findings suggest that ANXA6 potentiates M1 macrophage polarization through enhanced pyroptotic activation.

The molecular mechanisms governing macrophage polarization involve multiple signaling pathways, including STAT, NF-κB, and PPAR-γ-mediated transcriptional regulation 45. It was also shown that ROS are essential for maintaining the homeostasis of M1 phenotype macrophages and M2 phenotype macrophage cells under physiological conditions 46. In the present study, we found that ANXA6 can mediate macrophage M1 polarization and pyroptosis via the NF-κB/ROS signaling axis, thereby contributing to RSA pathogenesis.

Similarly, macrophages are critical in regulating trophoblast biological behavior 5, 30. The present results suggest that macrophages overexpressing ANXA6 inhibit trophoblast function, an effect that is mediated by the secretion of TNF-α. Moreover, our prior studies indicated that the PI3K/AKT signaling pathway is crucial for trophoblast growth, migration and invasion 4, 25, 47. In this study, our results further suggest that macrophages with overexpressed ANXA6 inhibit the PI3K/AKT axis through TNF-α, thereby repressing trophoblast function.

In conclusion, ANXA6 expression is significantly increased in decidual macrophages of RSA patients. Overexpressed ANXA6 significantly promoted ROS production and inhibited mitochondrial membrane potential, thereby promoting macrophage pyroptosis and M1 macrophage polarization, and the effects were mediated through the NF-κB/ROS axis. Subsequently, macrophages with ANXA6 overexpression yielded an inhibitory effect on trophoblast migration, invasion, and EMT *in vitro*. This effect was mediated by the inhibition of the PI3K/AKT axis via TNF-α. Finally, in an abortion mouse model, the ANXA6 expression level in decidual tissues was notably increased and highly correlated with pyroptosis. Further studies revealed that overexpression of ANXA6 in normal pregnant mice resulted in increased embryonic resorption, whereas knockdown of ANXA6 in abortion-prone mice alleviated embryonic resorption. Overall, our findings suggest that ANXA6 could be a potential target strategy for pregnancy-related diseases like RSA.

## Supplementary Material

Supplementary figures and tables.

## Figures and Tables

**Table 1:** Comparison of baseline data between the two groups of patients

Characteristic	HC (n=20)	RSA (n=20)	*P* value
Age (years)	29.75±3.16	30.55±2.33	0.37
BMI (kg/m^2^)	21.73±1.47	22.01±1.75	0.58
Gestation age (weeks)	7.45±0.83	7.70±1.03	0.40
Number of miscarriages	0.15±0.37	2.75±0.72	< 0.01
