# Supplementary material for: ANXA6 Overexpression Causes Abnormal Decidual Macrophage-Trophoblast Crosstalk in Recurrent Spontaneous Abortion
Source: Int J Biol Sci. 2025 Aug 11;21(12):5206–22. doi: 10.7150/ijbs.111791 (PMC12435268; doi:10.7150/ijbs.111791)
Supplement: Supplementary file 1 — Supplementary figures and tables. [file ijbsv21p5206s1.pdf]

Supplementary figures

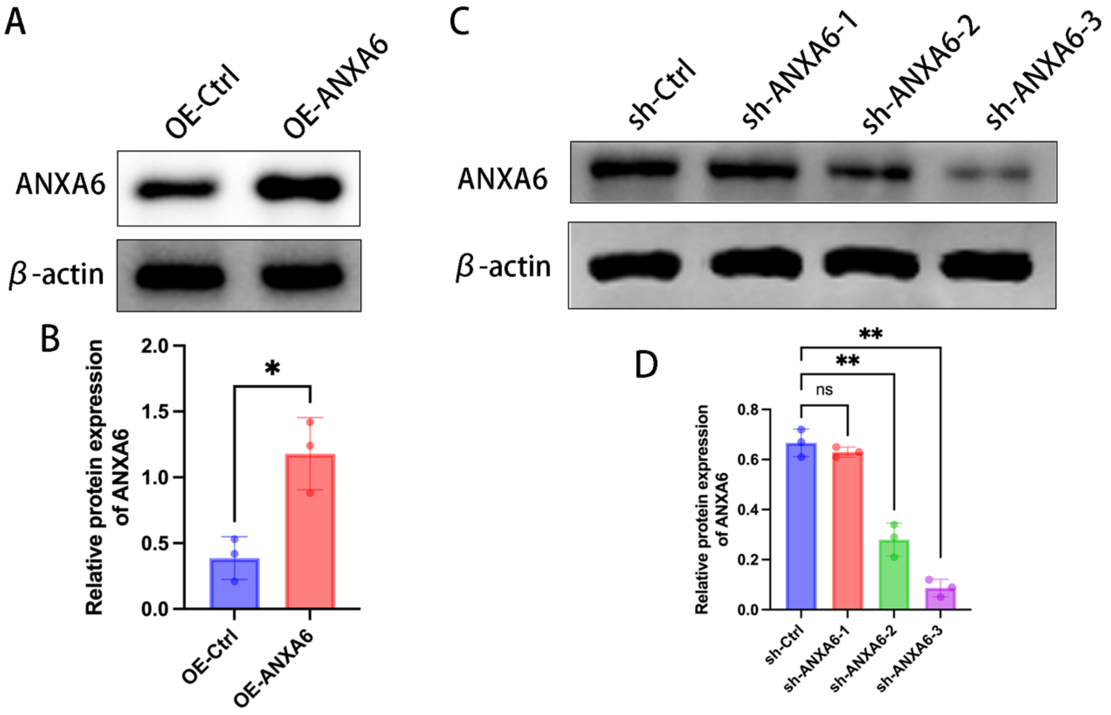

Figure S1. (A-D) Macrophages were infected with ANXA6 knockdown, ANXA6 overexpressing lentiviral vectors, and viral negative controls, and ANXA6 expression was assessed by western blotting, and the statistical value of ANXA6 was analyzed (n=3). Student's t-test was used to assess differences between the two groups, and one-way ANOVA was used to compare differences between multiple groups. *$P < 0.05$, **$P < 0.01$, ns: not significant.

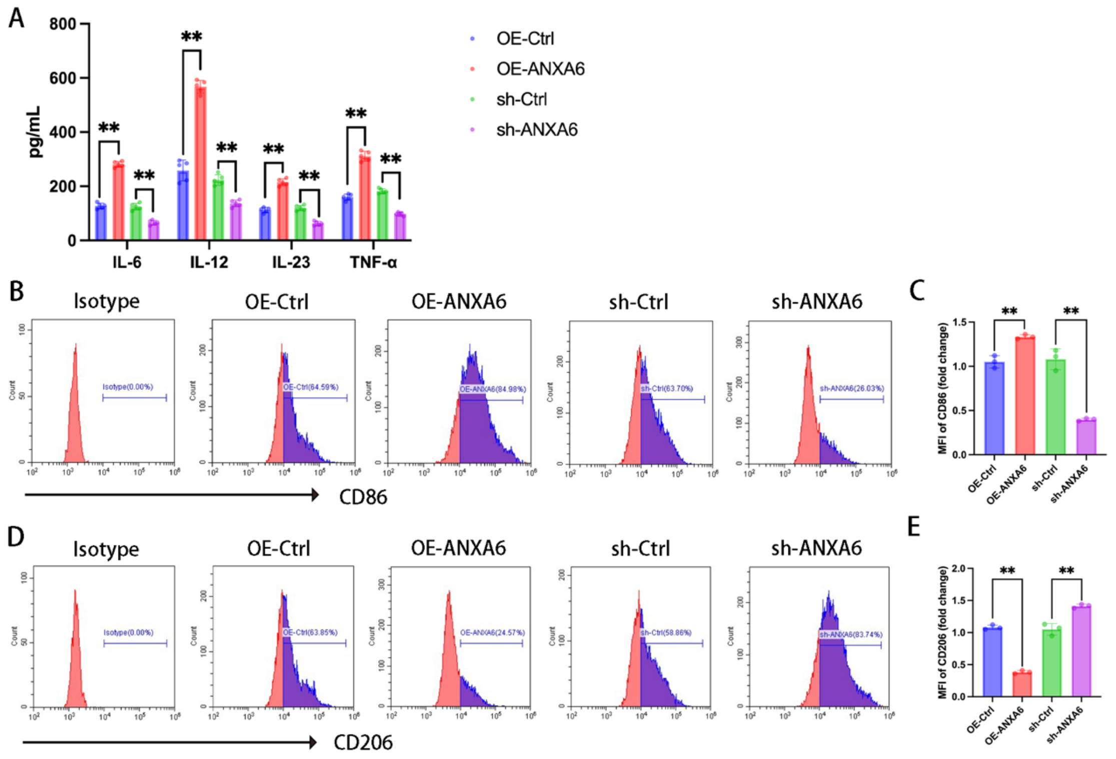

Figure S2. (A) Detection of inflammatory cytokine secretion by ELISA kits (n=5). **(B)** Representative flow cytometry results of CD86 expression in macrophages(n=3). **(C)** Quantitative flow cytometry results of CD86 expression in macrophages(n=3). **(D)** Representative flow cytometry results of CD206 expression in macrophages(n=3). **(E)** Quantitative flow cytometry results of CD206 expression in macrophages(n=3). One-way ANOVA was used to compare differences between multiple groups. $^{**}P < 0.01$.

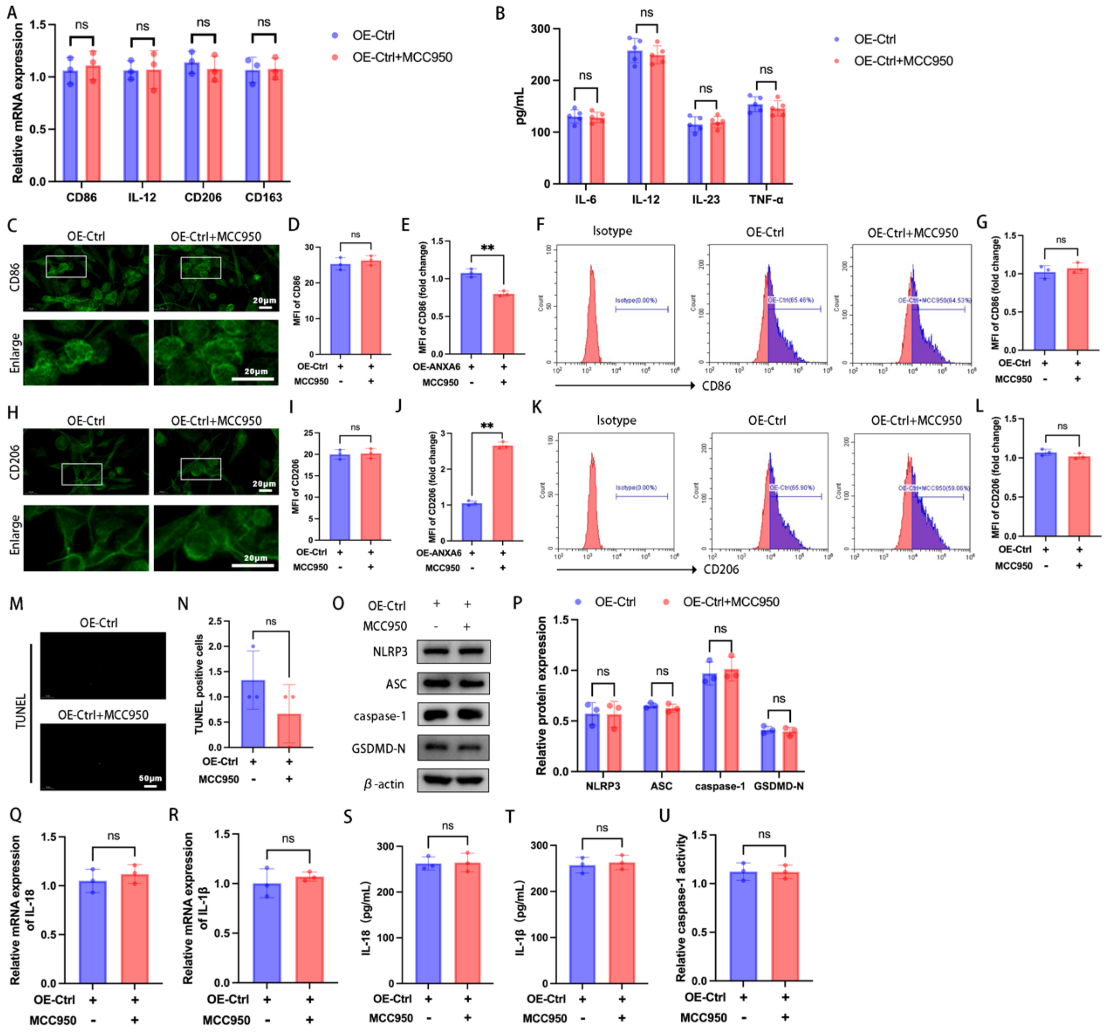

Figure S3. (A) The mRNA expression levels of CD86, IL-12, CD206, and CD163 were detected by qPCR (n=3). **(B)** Detection of inflammatory cytokine secretion by ELISA kits (n=5). **(C)** Representative fluorescence images of CD86(n=3). **(D)** Quantitative values of the MFI of CD86 (n=3). **(E)** Quantitative flow cytometry results of CD86 expression in macrophages(n=3). **(F)**Representative flow cytometry results of CD86 expression in macrophages(n=3). **(G)** Quantitative flow cytometry results of CD86 expression in macrophages(n=3). **(H)** Representative fluorescence images of CD206(n=3). **(I)** Quantification of the MFI of CD206 (n=3). **(J)** Quantitative flow cytometry results of CD206 expression in macrophages (n=3). **(K)** Representative flow cytometry results of CD206 expression in macrophages (n=3). **(L)** Quantitative flow cytometry results of CD206 expression in macrophages (n=3). **(M)** Examination of pyroptosis of each group

of cells by TUNEL staining (n=3). **(N)** Statistical value of the number of TUNEL-positive cells (n=3). **(O-P)** The expression of NLRP3, ASC, caspase-1 and GSDMD-N was determined by western blotting (n=3), and the statistical value of NLRP3, ASC, caspase-1, and GSDMD-N was analyzed. **(Q-R)** The expression of inflammatory cytokines IL-1β and IL-18 was detected by qPCR (n=3). **(S-T)** The expression of inflammatory cytokines IL-1β and IL-18 was detected using ELISA kits (n=3). **(U)** The activity of caspase-1 (n=3). Student's t-test was used to assess differences between the two groups. ** $P < 0.01$, ns: not significant.

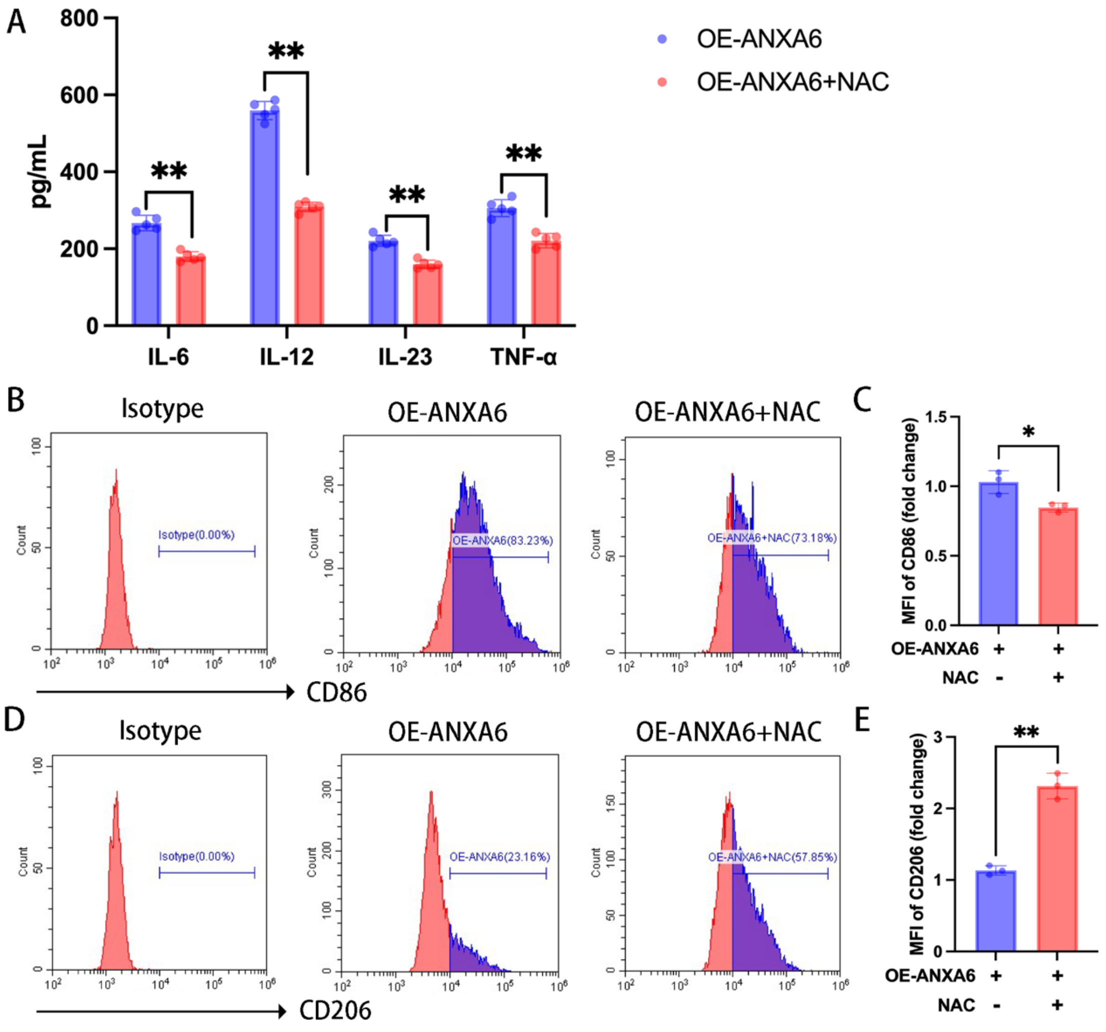

Figure S4. (A) Detection of inflammatory cytokine secretion by ELISA kits (n=5). **(B)** Representative flow cytometry results of CD86 expression in macrophages(n=3). **(C)** Quantitative

flow cytometry results of CD86 expression in macrophages(n=3). **(D)** Representative flow cytometry results of CD206 expression in macrophages(n=3). **(E)** Quantitative flow cytometry results of CD206 expression in macrophages(n=3). Student's t-test was used to assess differences between the two groups. *$P < 0.05$, **$P < 0.01$.

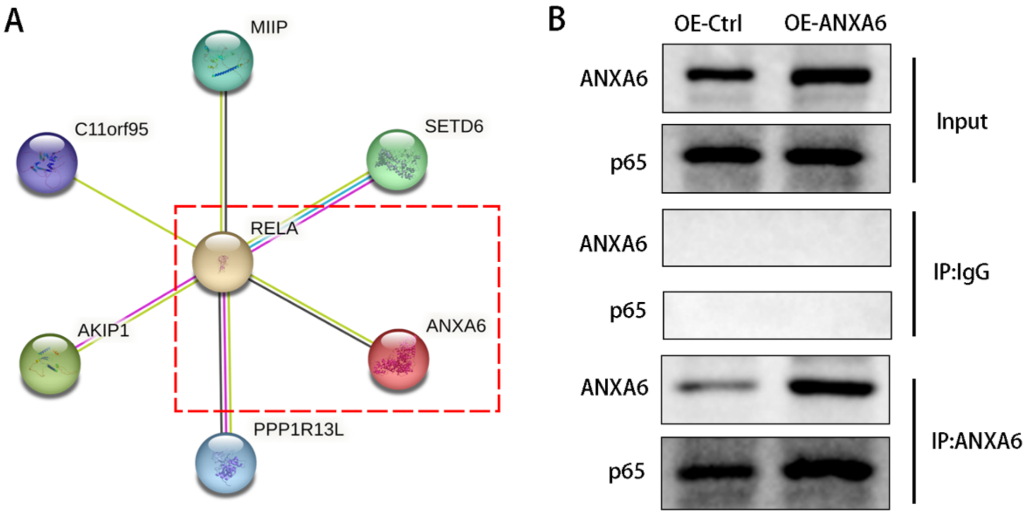

Figure S5. (A) The protein-protein interaction network of RELA and ANXA6. **(B)** Representative Co-immunoprecipitation images(n=3).

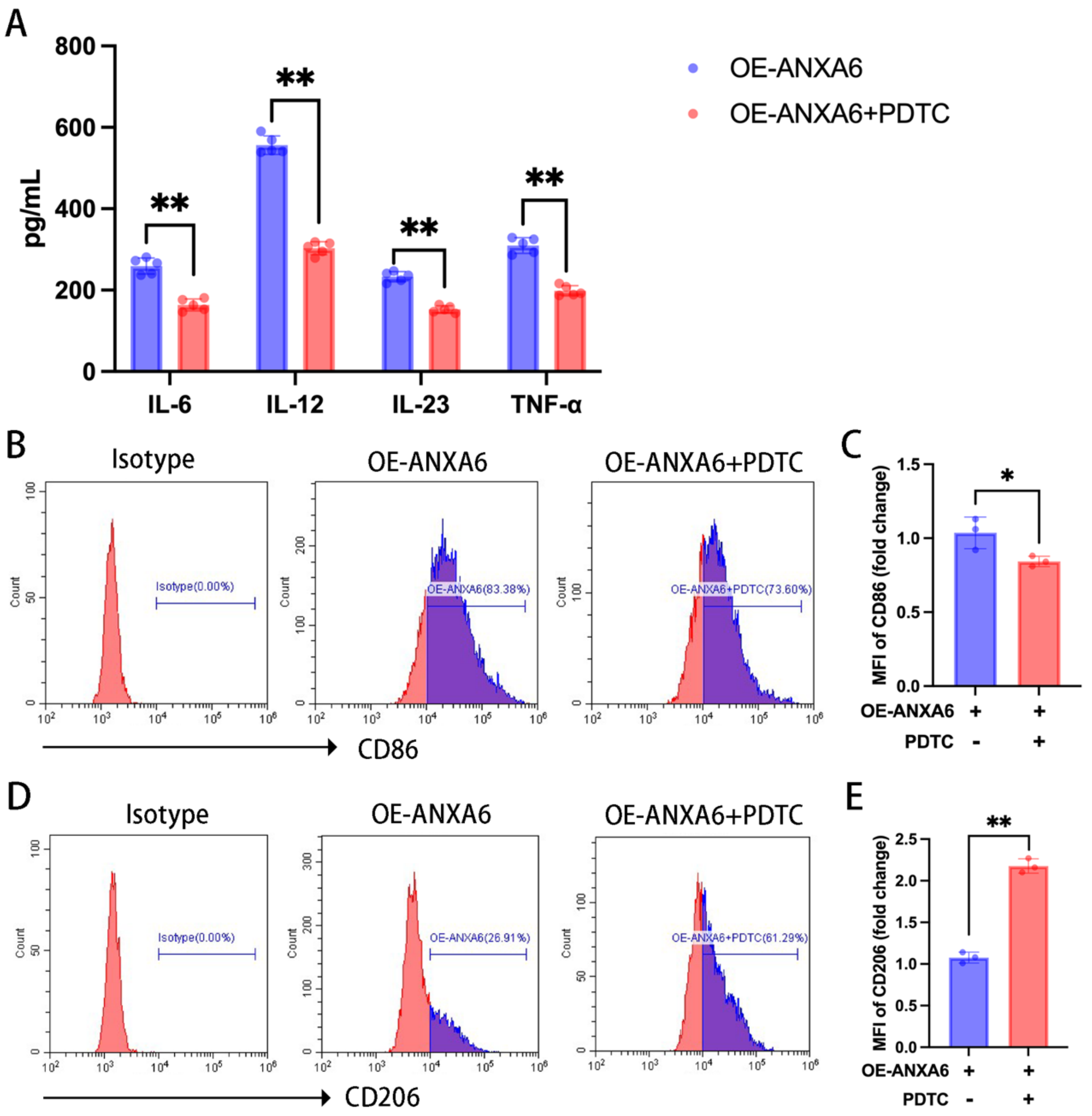

Figure S6. (A) Detection of inflammatory cytokine secretion by ELISA kits (n=5). **(B)** Representative flow cytometry results of CD86 expression in macrophages(n=3). **(C)** Quantitative flow cytometry results of CD86 expression in macrophages(n=3). **(D)** Representative flow cytometry results of CD206 expression in macrophages(n=3). **(E)** Quantitative flow cytometry results of CD206 expression in macrophages(n=3). Student's t-test was used to assess differences between the two groups. *$P < 0.05$, **$P < 0.01$.

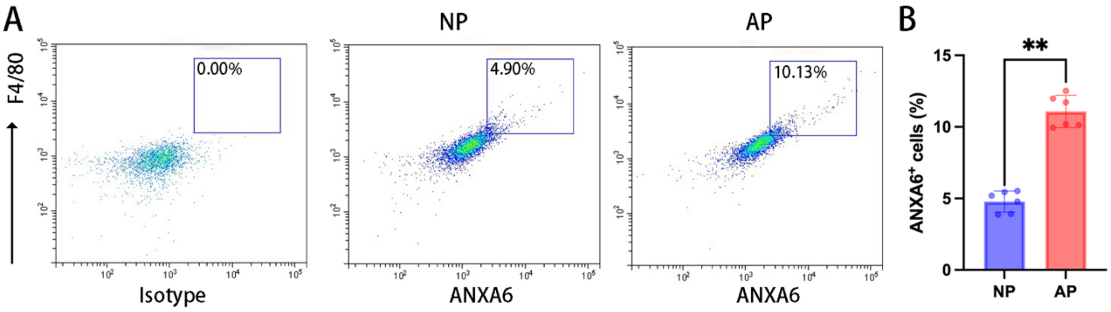

Figure S7. (A-B) Representative and quantitative flow cytometry results for ANXA6 expression in F4/80+ decidual macrophages (n=6). Student's t-test was used to assess differences between the two groups. $**P < 0.01$.

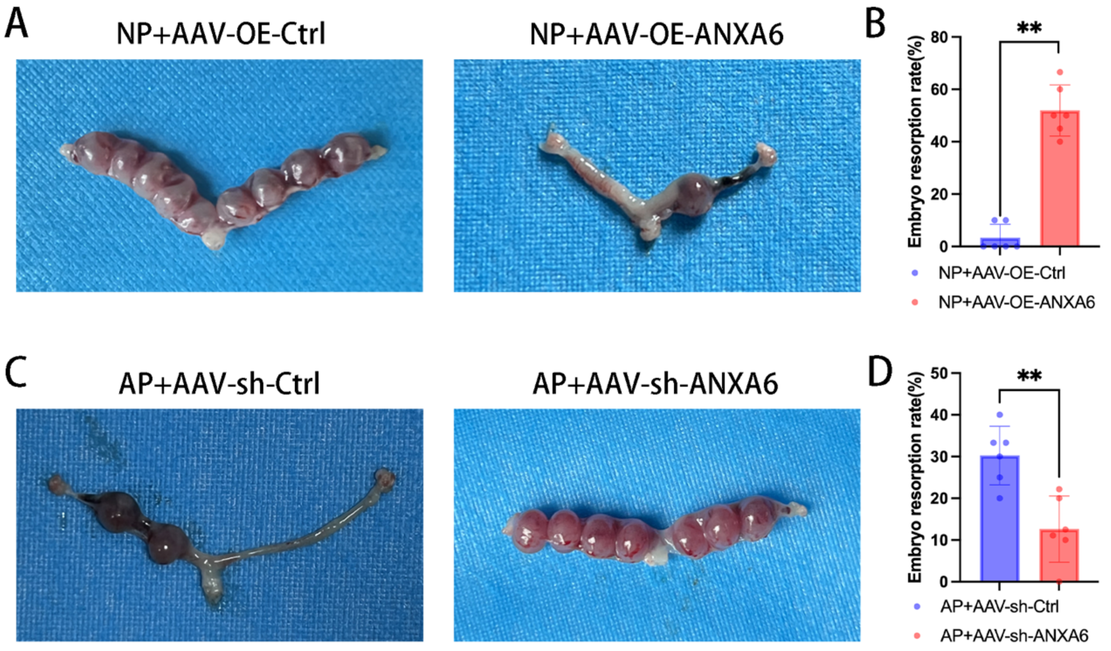

Figure S8. (A-B) NP mice were treated with AAV-OE-ANXA6 or AAV-OE-Ctrl, and embryo resorption rates were measured at day 11.5 of gestation (n=6). **(C-D)** AP mice were treated with AAV-sh-ANXA6 or AAV-sh-Ctrl, and embryo resorption rates were measured at day 11.5 of gestation (n=6). A Student's t-test was used to assess the differences between the two groups. $**P < 0.01$.

Supplementary tables

Table S1. The primer sequences used in the study.

Primer	Forward	Reverse
Human-ANXA6	AGAGCTACAAGTCCCTCTACG	CCCACAATCAACCGTTCAAAC
Human-CD86	CTGCTCATCTATACACGGTTACC	GGAAACGTCGTACAGTTCTGTG
Human-IL-12	CCTTGCACTTCTGAAGAGATTGA	ACAGGGCCATCATAAAAGAGGT
Human-CD206	TCCGGGTGCTGTTCTCCTA	CCAGTCTGTTTTTGATGGCACT
Human-CD163	TTTGTCAACTTGAGTCCCTTCAC	TCCCGCTACACTTGTTTTCAC
Human-IL-18	TCTTCATTGACCAAGGAAATCGG	TCCGGGGTGCATTATCTCTAC
Human-IL-1β	ATGATGGCTTATTACAGTGGCAA	GTCGGAGATTCGTAGCTGGA
Human-IL-33	GCCTGTCAACAGCAGTCTACTG	TGTGCTTAGAGAAGCAAGATACTC
Human-CXCL-1	AGCTTGCCTCAATCCTGCATCC	TCCTTCAGGAACAGCCACCAGT
Human-G-CSF	ATAGCGGCCTTTTCCTCTACC	GCCATTCCCAGTTCTTCCAT
Human-Wnt5a	ATTCTTGGTGGTCGCTAGGTA	CGCCTTCTCCGATGTACTGC
Human-TNF-α	TCTCGAACCCCGAGTGACAA	TGAAGAGGACCTGGGAGTAG
Human-β-actin	CATGTACGTTGCTATCCAGGC	CTCCTTAATGTCACGCACGAT
Mouse-ANXA6	CACAGGGTGCCATGTACCG	GTCCTTGCCATACAGGGACTT
Mouse-β-actin	GTGACGTTGACATCCGTAAAGA	GCCGGACTCATCGTACTCC

Table S2. The specific antibodies used for Western blotting, Co-IP, immunohistochemistry, immunofluorescence staining, and flow cytometry.

Antibodies	Identifier	Source
Anti-ANXA6	12542-1-AP	Proteintech
Anti-ANXA6	ab201024	Abcam
anti-NLRP3	30109-1-AP	Proteintech
anti-ASC	10500-1-AP	Proteintech

anti-caspase-1	22915-1-AP	Proteintech
anti-GSDMD-N	DF13758	Affinity Biosciences
anti-p65	80979-1-RR	Proteintech
anti-p-p65	AF2006	Affinity Biosciences
anti-p-PI3K	AF3242	Affinity Biosciences
anti-PI3K	20584-1-AP	Proteintech
anti-p-AKT	AF0016	Affinity Biosciences
anti-AKT	10176-2-AP	Proteintech
anti-β-actin	66009-1-Ig	Proteintech
HRP-conjugated Goat Anti-Mouse IgG(H+L)	SA00001-1	Proteintech
HRP-conjugated Goat Anti-Rabbit IgG(H+L)	SA00001-2	Proteintech
anti-CD68	28058-1-AP	Proteintech
anti-CD86	ab239075	Abcam
anti-CD206	ab64693	Abcam
anti-F4/80	30325	CST
anti-E-cadherin	20874-1-AP	Proteintech
anti-N-cadherin	22018-1-AP	Proteintech
anti-Vimentin	10366-1-AP	Proteintech
HRP labeled goat anti-rabbit	5220-0336	SeraCare
PE Anti-human CD206 Antibody	321105	BioLegend
PE Anti-human CD86 Antibody	374206	BioLegend
PE Anti-Mouse F4/80 Antibody	E-AB-F0995D	Elabscience
Goat Anti-Rabbit IgG H&L (Alexa Fluorr® 488)	ab150077	Abcam
